# Supplementary material for: A genome-wide CRISPR screen in Anopheles mosquito cells identifies fitness and immune cell function-related genes
Source: Nat Commun. 2025 Nov 24;16:10323. doi: 10.1038/s41467-025-65304-y (PMC12644648; doi:10.1038/s41467-025-65304-y)
Supplement: Supplementary file 1 — Supporting Information [file 41467_2025_65304_MOESM1_ESM.pdf]

Supporting Information

A genome-wide CRISPR screen in *Anopheles* mosquito cells identifies fitness and immune cell function-related genes

Enzo Mameli[1#], George-Rafael Samantsidis[2#], Raghuvir Viswanatha[1], Hyeogsun Kwon[2], David R. Hall[2], Matthew Butnaru[1], Yanhui Hu[1], Stephanie E. Mohr[1], Norbert Perrimon[1,3*], Ryan C. Smith[2*]

[1]Department of Genetics, Blavatnik Institute, Harvard Medical School, Boston, MA, 02115, USA

[2]Department of Plant Pathology, Entomology and Microbiology, Iowa State University, Ames, IA 50011, USA

[3]HHMI, Harvard Medical School, Boston, MA, 02115, USA

[#]These authors contributed equally

*These authors jointly supervised this work

Email: perrimon@genetics.med.harvard.edu; smithr@iastate.edu

Included supporting information

Supplementary Figures

Supplementary Fig. 1. Comparison of scores in for replicates 1 and 2 of the genome-wide *Anopheles* CRISPR knockout screen for fitness-related genes.

Supplementary Fig. 2. Gene set enrichment analysis of *Drosophila* orthologs of *Anopheles* essential genes using the PANGEA online resource.

Supplementary Fig. 3. *Serpent*-silencing influences mosquito immune cell numbers and malaria parasite infection.

Supplementary Fig. 4. Gene set enrichment analysis of *Drosophila* orthologs of *Anopheles* clodronate resistance screen results using the PANGEA online resource.

Supplementary Fig. 5. Evaluation of clodronate liposome-mediated phagocyte depletion at different concentrations and over time.

Supplementary Fig. 6. Timing of liposome uptake and immune cell depletion.

Supplementary Fig. 7. Day 4 RNAi of candidate genes.

Supplementary Fig. 8. Examinations of RNAi efficiency based on hemocyte enrichment.

Supplementary Tables

Supplementary Table 1. Makeup of the genome-wide sgRNA library used in this study.

Supplementary Table 2. Primers used for dsRNA synthesis.

Supplementary Table 3. Primers used for gene expression analysis.

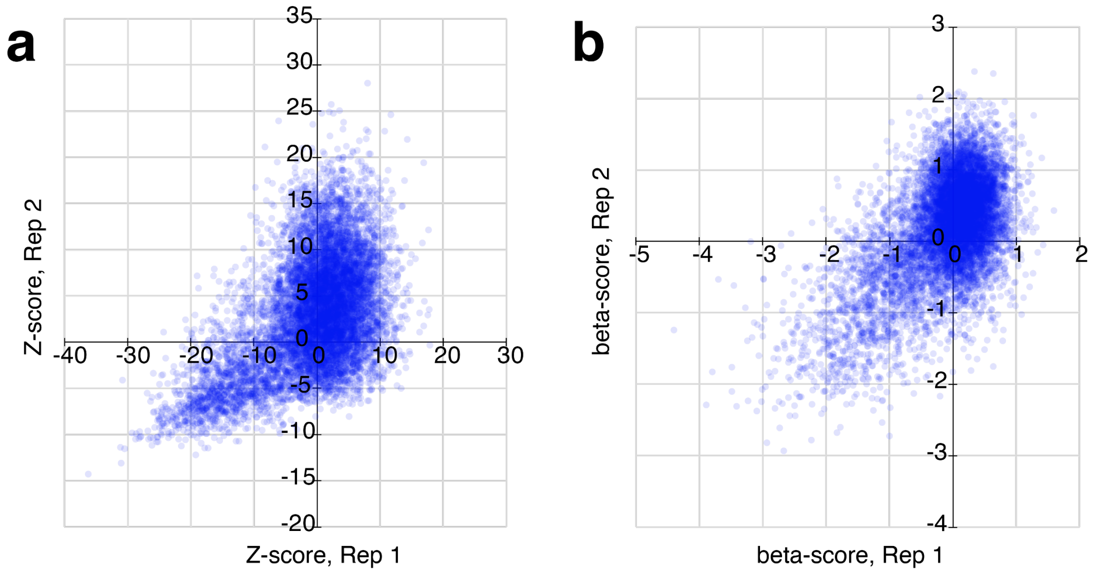

Supplementary Fig. 1. Comparison of scores in for replicates 1 and 2 of the genome-wide *Anopheles* CRISPR knockout screen for fitness-related genes. Correlations between (**a**) gene-level Z-scores (R^2 = 0.25) or (**b**) gene-level beta-scores (R^2 = 0.33) for each of the two replicates. The observation that the beta scores are more closely correlated than the Z-scores might be due to a difference in background noise between replicates. Source data are provided as a Source Data file.

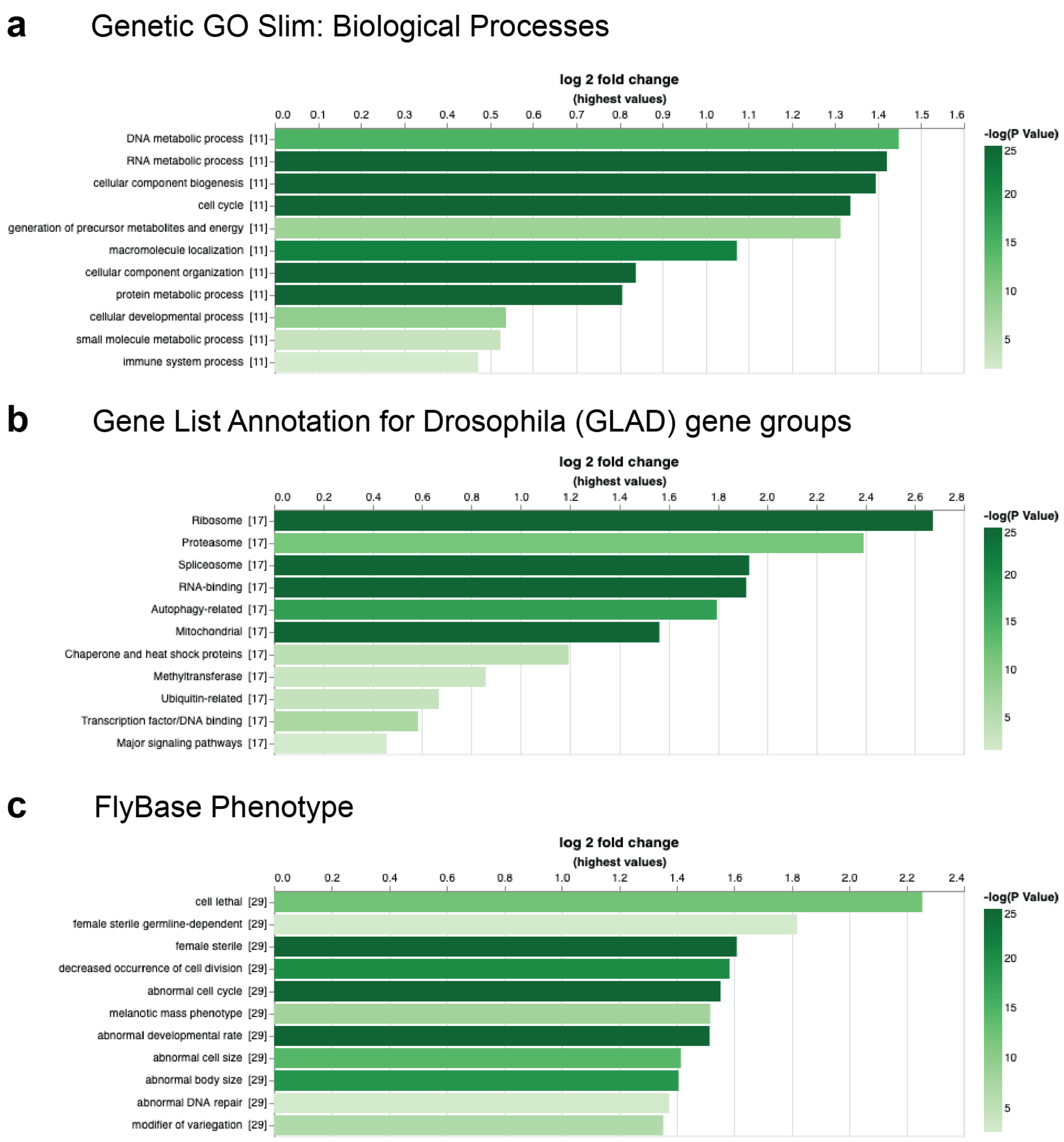

Supplementary Fig. 2. Gene set enrichment analysis of *Drosophila* orthologs of *Anopheles* essential genes using the PANGEA online resource. Genes that scored as fitness genes in replicate 1 of the genome-wide *Anopheles* cell screen were mapped to *Drosophila* orthologs using DIOPT. Only the top-scoring ortholog match was selected; if more than one *Drosophila* gene had the same DIOPT ortholog score, we arbitrarily chose one of the genes for the analysis. The *Drosophila* gene list was then used as the

input at PANGEA. (**a**) Enrichment of gene ontology (GO) slim “Biological Process” annotations (Gene Ontology sets>Gene Ontology Subsets>Generic GO consortium Subsets (GO slim)>SLIM1 GO BP). (**b**) Enrichment of Gene List Annotation for Drosophila (GLAD) gene sets (Other Gene sets>DRSC GLAD Gene Group). (**c**) Enrichment of FlyBase phenotype annotations for classical mutations (Other Gene sets>Phenotype>FlyBase phenotype for classical alleles). In each case, the top ten results as ranked based on fold-change are displayed. Length of the bars represents fold enrichment of genes within each category while darkness reflects the *P* value. Only GO terms with *P* value below the 0.05 threshold are displayed, up to a maximum of 10 terms. The full PANGEA analysis outputs including results of multiple statistical analyses are included in **Supplementary Data 4**.

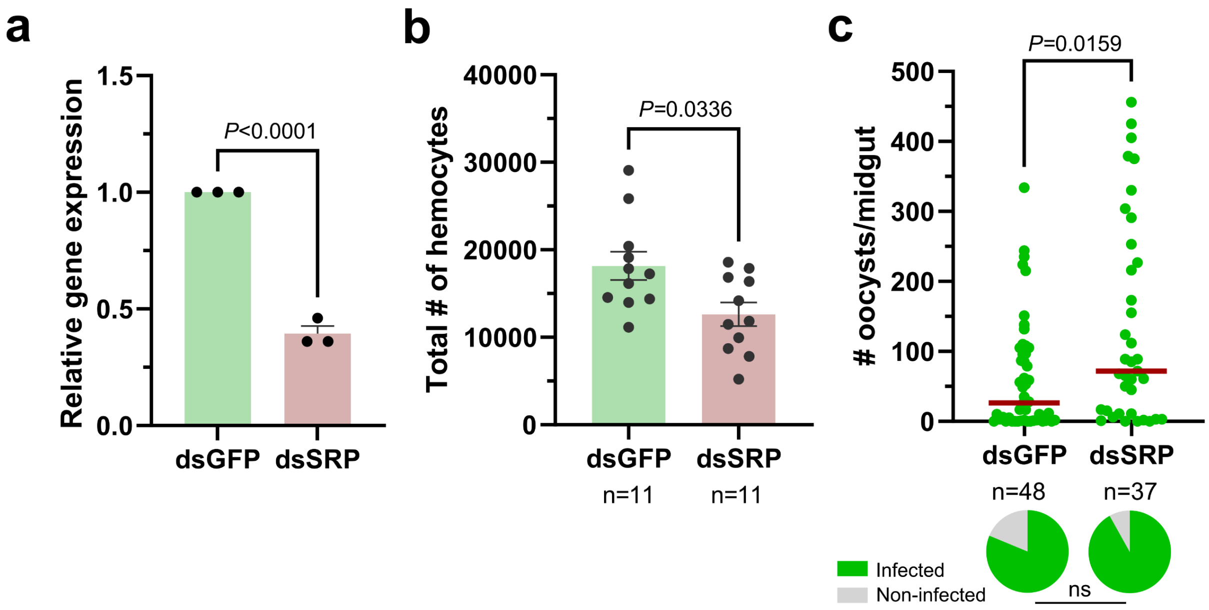

Supplementary Fig. 3. *Serpent*-silencing influences mosquito immune cell numbers and malaria parasite infection. (**a**) Confirmation of *serpent* (*srp*) gene-silencing by qRT-PCR. Data were collected from three independent experiments of ~10 adult female mosquitoes at two days post-dsRNA injection. Statistical analysis was performed using a two-tailed unpaired t-test. Exact *P* values are displayed in the figure. (**b**) Examinations of total hemocyte counts from individual mosquitoes (n=11) were performed in control (*dsGFP*) and *srp*-silenced backgrounds. Data are displayed from two independent experiments, with statistical analysis performed using a two-tailed Mann-Whitney test. Exact *P* values are displayed in the figure. (**c**) The effects of *srp*-silencing on *P. berghei* infection were evaluated by examining oocyst numbers from individual mosquitoes (dots, n) at 10 days post-infection. Median oocyst numbers from three independent biological replicates are indicated by the horizontal red line. Data were analyzed using a two-tailed Mann-Whitney test, exact *P* values displayed in the figure. The prevalence of infection (% infected/total) is depicted for mosquitoes in each experimental condition and examined using a two-tailed Fisher's exact test to determine significance. ns, not significant. Additional details of the statistical analysis for **a**-**c** are included in the Source Data file. Source data are provided as a Source Data file.

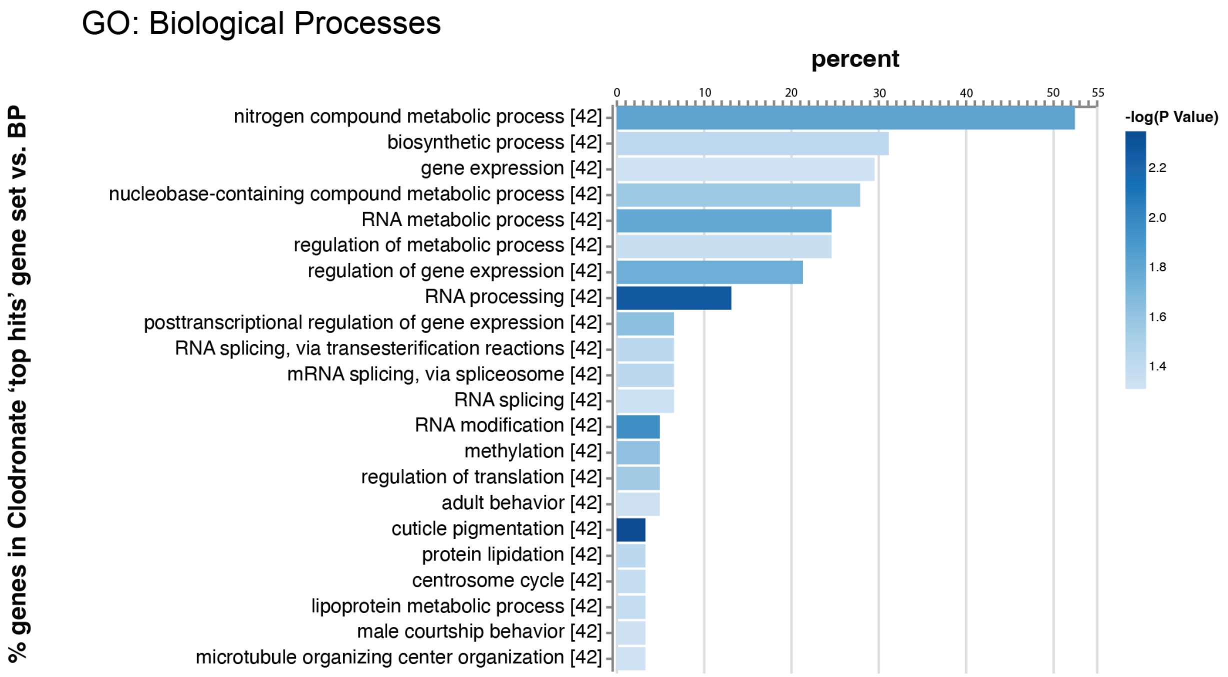

Supplementary Fig. 4. Gene set enrichment analysis of *Drosophila* orthologs of *Anopheles* clodronate resistance screen results using the PANGEA online resource. Genes that scored in the genome-wide *Anopheles* cell screen for resistance to clodronate treatment were mapped to *Drosophila* orthologs using DIOPT. Only the top-scoring ortholog match was selected; if more than one *Drosophila* gene had the same DIOPT ortholog score, we arbitrarily chose one of the genes for the analysis. The *Drosophila* gene list was then used as the input at PANGEA. Only significantly enriched gene sets ($P<0.05$) are shown for enrichment of GO terms for *Drosophila* as reported by FlyBase and the Alliance of Genome Resources (Gene Ontology sets> GO Biological Process). The enriched gene sets are ranked based on the percentage of genes in the clodronate screen gene set vs. the GO Biological Process gene set. Length of the bars represents percentage of genes within each category while darkness reflects the *P* value calculated on the GO enrichment. The full PANGEA analysis outputs including results of multiple statistical analyses are included in **Supplementary Data 4**.

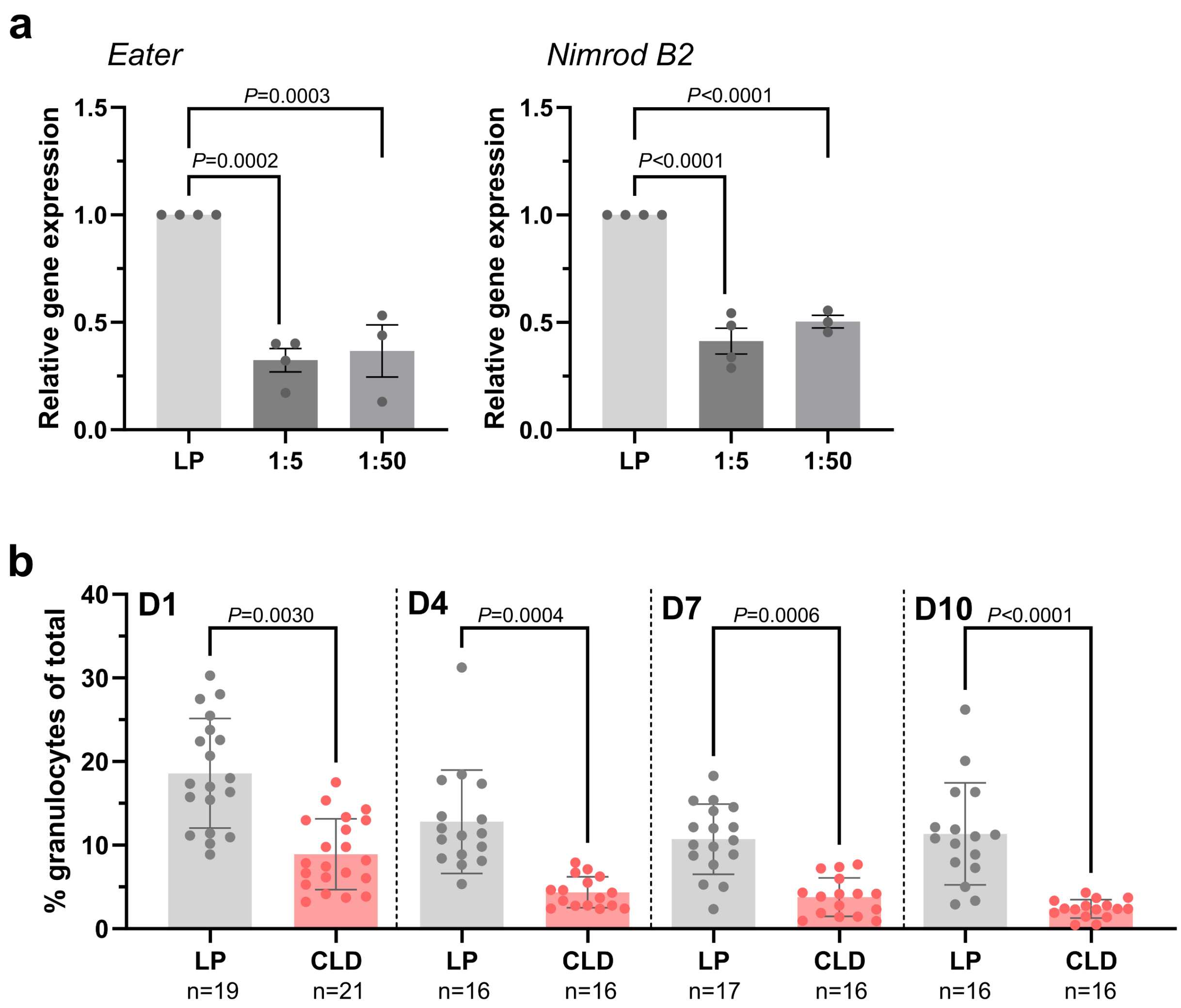

Supplementary Fig. 5. Evaluation of clodronate liposome-mediated phagocyte depletion at different concentrations and over time. (**a**) The expression of *eater* and *Nimrod B2* was evaluated by qRT-PCR in mosquitoes injected with 1:5 or 1:50 dilutions of clodronate liposomes and compared to empty liposome controls (LP). Pooled mosquitoes from three or more independent experiments were examined at 24 hours post-injection using a one-way ANOVA and a Holm-Sidak's multiple comparison test. Exact *P* values are displayed in the figure where significant. (**b**) The duration of phagocytic granulocyte depletion was examined at 1, 4, 7, and 10 days (D) following the injection of either control or clodronate liposomes (1:50 dilution). The proportion of granulocytes of the total hemocyte population from was quantified in individual mosquitoes (n) via a hemocytometer following hemolymph perfusion. Data were pooled from two or more

independent experiments and analyzed using a two-tailed Mann-Whitney test to determine significance. Exact *P* values are displayed in the figure. Additional details of the statistical analysis in **a** and **b** are included in the Source Data file. Source data are provided as a Source Data file.

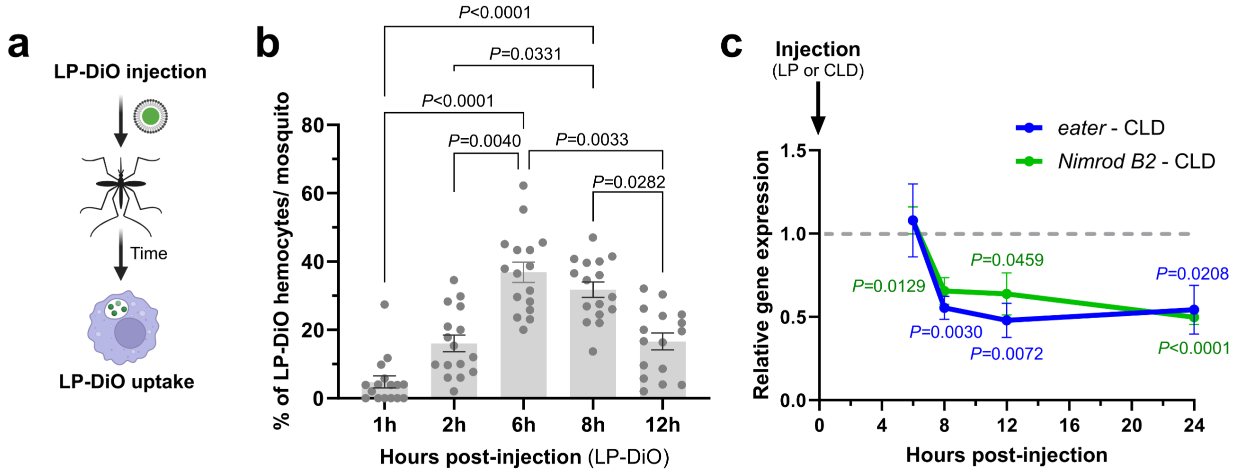

Supplementary Fig. 6. Timing of liposome uptake and immune cell depletion. (**a**) Experimental overview of methodology used to examine the timing of liposome (LP-DiO) uptake in vivo by circulating immune cells. (**b**) The percentage of LP-DiO$^+$ cells at 1, 2, 6, 8, or 12 hours post-injection. Each dot corresponds to data collected from an individual mosquito (n=16 for all timepoints; pooled from two independent replicates). Data were analyzed using Kruskal-Wallis and Dunn's multiple comparison test to determine significance. Adjusted *P* values are displayed in the figure where significant. (**c**) The timing of clodronate liposome-mediated cell ablation was determined *in vivo* by measuring *eater* and *Nimrod B2* expression at 6, 8, 12, and 24 hours post-injection of control or clodronate liposomes in adult female mosquitoes. Data are displayed as the mean ±SEM from three or more independent experiments were analyzed using a two-tailed unpaired t test to compare expression levels between control- or clodronate liposome-treated mosquitoes. Exact *P* values are displayed in the figure where significant. Additional details of the statistical analysis in **b** and **c** are included in the Source Data file. Illustrations in **a** were created in part using BioRender. Smith, R. (2025) https://BioRender.com/0t47etj and images created by David Hall using Inkscape. Source data are provided as a Source Data file.

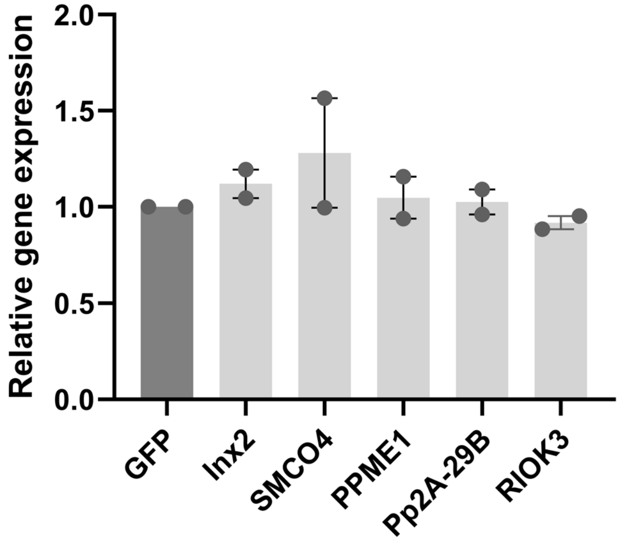

Supplementary Fig. 7. Day 4 RNAi of candidate genes. For candidate genes where RNAi was unsuccessful at two days post-injection, additional RNAi experiments were performed in which knockdowns were evaluated at four days post-injection. Expression data from two independent experiments are displayed as the mean ±SEM and compared to GFP controls. The dark shaded bar denotes the control. Source data are provided as a Source Data file.

a **Hemocyte enrichment** (Raddi *et al.* 2020)

Gene ID	Hemocytes	Non-hemocytes	RNAi?
AGAP001488	-	-	-
AGAP002257	Y	-	Y
AGAP002672	Y	-	Y
AGAP003004	-	-	Y
AGAP003534	-	Y	-
AGAP004382	-	Y	Y
AGAP008336	-	-	-
AGAP008757	-	-	Y
AGAP009105	-	-	-
AGAP009993	-	-	-

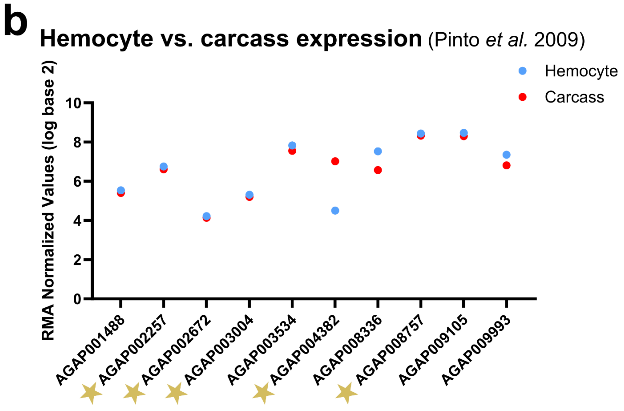

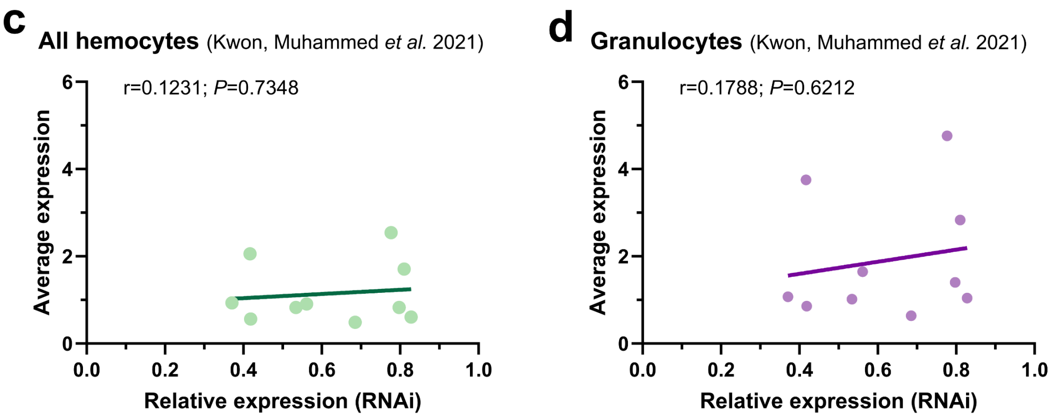

Supplementary Fig. 8. Examinations of RNAi efficiency based on hemocyte enrichment. Following variability in the success of RNAi for our candidate gene targets, we first examined the enrichment of these genes in hemocytes and non-hemocyte tissues using data from previously published studies (**a** and **b**). (**a**) Analysis from Raddi *et al.* (2020) displays the significant enrichment of candidate genes in either hemocyte or non-hemocyte tissues and whether the gene target is silenced via RNAi. (**b**) Expression data from Pinto *et al.* (2009) display normalized values for hemocyte and carcass tissues for each candidate gene. Successfully silenced genes are denoted by the gold star. Additional analysis using expression data from Kwon, Muhammed *et al.* (2021) examines correlations of gene-silencing with gene expression averaged from (**c**) all hemocyte subtypes or from (**d**) granulocyte populations (presumed target of clodronate-mediated depletion). For both **c** and **d**, lines display the best-fit line, with r and *P* values displayed resulting Pearson's correlation analysis. Additional details of the statistical analysis for **c** and **d** are included in the Source Data file. Source data are provided as a Source Data file.

Supplementary Table 1. Makeup of the genome-wide sgRNA library used in this study.

**Guide type or target**	**Number of guides (number of genes)**
Targeting intergenic regions (negative controls)*	400 sgRNAs
Non-targeting (negative controls)	100 sgRNAs
Positive controls (based on Viswanatha, Mameli, et al. 2021 *Nature Communications*)	461 sgRNAs (3 genes)
Protein-coding genes (total unique)	87,812 sgRNAs (12,774 genes)<br>• 12,323 genes covered by 7 sgRNAs/gene<br>• 301 genes covered by 3-6 sgRNAs/gene<br>• 150 genes covered by 1-2 sgRNAs/gene
Non-protein-coding genes (total unique)	951 sgRNAs (230 genes)<br>• 57 genes covered by 7 sgRNAs/gene<br>• 104 genes covered by 3-6 sgRNAs/gene<br>• 69 genes covered by 1-2 sgRNAs/gene
Duplicated designs*	484 sgRNAs (235 genes)
Total unique designs	89,724 sgRNAs
Total sgRNAs including duplications	90,208 sgRNAs

* We included two copies of one sgRNA design for 71 genes for which only 1 unique sgRNA per gene met our design criteria, 79 genes for which only 2 sgRNAs met criteria, and 85 genes for which only 3 sgRNAs met criteria.

** Intergenic control sgRNAs were selected in regions located at least 10 kb away from any annotated genes, including protein-coding genes, lncRNAs, and other known transcript types. However, we cannot fully exclude the possibility that some of these sgRNAs may fall within unannotated regulatory regions (e.g., long-distance enhancers), as such features are not well-annotated in mosquitoes.

Supplementary Table 2. Primers used for gene expression analysis.

Gene ID	Annotation	Primer qPCR-F	Primer qPCR-R
AGAP001488	Inx2	ATGCGGTTCTTCTTCTGCGA	TGGTCACCTTGGGAAAGACG
AGAP002238	Srp	CAAGATGAACGGCATGAATC	CTGGTTCACCGACTTGATTG
AGAP002257	Tsp3A	AACATCATGTGCGGCTACCA	TCGAGCGTTTTGGCGAGATA
AGAP002672	TMEM8B	CAGCACCACAAGTACGCAAC	AGGCAGGTGGAAAACACGAA
AGAP003004	Traf6	GCACGGGTACAAGTTTTGCG	CTTGATGCGTCCCTTGAACG
AGAP003534	SMCO4	GCGGTGGACAGACGAAAGAAAC	ATCGTGGGCGAGTTTTCAGA
AGAP004382	GSTD3	GCCTCATTTCACCGCCCTG	CTTCAGGTTCAGCGTAATGCC
AGAP008336	PPME1	GAGTTTCCTCCGATCCCGTC	TTTTCCTCCGACGAGCCTTC
AGAP008757	TMEM147	CACTTTGGTAACTGTGCCGC	AACGCATTTCCAGAACGCAC
AGAP009105	PP2A-29B	ACCTGATCCCCACCTTCGTA	CAGTTCCACCTCGTCCTGTG
AGAP009993	RIOK3	ACGGTGGATGACGATATGCC	CGACGCTAAGTACCTCCTCC
AGAP010592	rpS7	ACCACCATCGAACACAAAGTTGACACT	CTCCGATCTTTCACATTCCAGTAGCAC
AGAP012386	eater	TTCACCCGTCTGCGAGGGATGCAAGC	GTCAACGTGCATAGTAGCGTCTCCGTAGC
AGAP029054	Nimrod B2	CAATCTGCTCAAATGGCTGCTTCCACG	GCTGCAAACATTCGGTCCAGTGCATTC

Supplementary Table 3. Primers used for dsRNA synthesis.

Gene ID	Annotation	Primer T7 F	Primer T7R
AGAP001488	Inx2	taatacgactcactatagggCAGGTTCCTTCGTAACCAGC	taatacgactcactatagggGACGACACTGAGTCTCTCGCT
AGAP002238	Srp	taatacgactcactatagggGTACTACAAACTGCACAACGTGAAC	taatacgactcactatagggCTGGTTGTTATATTTGCTGTTGCTG
AGAP002257	Tsp3A	taatacgactcactatagggTTTCCGTAGGCACAGATTCG	taatacgactcactatagggGTTCCGTTGCCTGTTGTCCT
AGAP002672	TMEM8B	taatacgactcactatagggGGCGTTGTTACCCTCACACT	taatacgactcactatagggGTGCAGCGTAATATCGTTCG
AGAP003004	Traf6	taatacgactcactatagggCAGAGTGCCGCTAAAGCTGA	taatacgactcactatagggCTTCATCCGGAGCAACAAAC
AGAP003534	SMCO4	taatacgactcactatagggTCTCGCTAATCTTGAAGGGG	taatacgactcactatagggCAAATAACCGATTTGTGCATGT
AGAP004382	GSTD3	taatacgactcactatagggCGCCACCCATCCCTTAAT	taatacgactcactatagggACGTCGTGTGGGAATCGTA
AGAP008336	PPME1	taatacgactcactatagggAGCGGTCGATGTAGCAAAAG	taatacgactcactatagggACTTCACTGCTGAAATGAGCC
AGAP008757	TMEM147	taatacgactcactatagggGGGCGGAATTTACGTGTTTA	taatacgactcactatagggCACCAGCTTCCTGTCCAGAT
AGAP009105	PP2A-29B	taatacgactcactatagggGCCAAGGTGACCGAGTTCT	taatacgactcactatagggCAGCGGCATGTACTCGATG
AGAP009993	RIOK3	taatacgactcactatagggAACCCGTAGCGGAGGTAGAT	taatacgactcactatagggGGAATCACGCGATAGTTTTTG
