# Supplementary material for: A genome-wide CRISPR screen in Anopheles mosquito cells identifies fitness and immune cell function-related genes
Source: Nat Commun. 2025 Nov 24;16:10323. doi: 10.1038/s41467-025-65304-y (PMC12644648; doi:10.1038/s41467-025-65304-y)
Supplement: Supplementary file 3 — Reporting Summary [file 41467_2025_65304_MOESM3_ESM.pdf]

nature portfolio

Corresponding author(s): Smith, Ryan C.

Last updated by author(s): Sep 30, 2025

Reporting Summary

Nature Portfolio wishes to improve the reproducibility of the work that we publish. This form provides structure for consistency and transparency in reporting. For further information on Nature Portfolio policies, see our Editorial Policies and the Editorial Policy Checklist.

Statistics

For all statistical analyses, confirm that the following items are present in the figure legend, table legend, main text, or Methods section.

n/a	Confirmed	
☐	☒	The exact sample size (*n*) for each experimental group/condition, given as a discrete number and unit of measurement
☐	☒	A statement on whether measurements were taken from distinct samples or whether the same sample was measured repeatedly
☐	☒	The statistical test(s) used AND whether they are one- or two-sided<br>*Only common tests should be described solely by name; describe more complex techniques in the Methods section.*
☐	☒	A description of all covariates tested
☐	☒	A description of any assumptions or corrections, such as tests of normality and adjustment for multiple comparisons
☐	☒	A full description of the statistical parameters including central tendency (e.g. means) or other basic estimates (e.g. regression coefficient) AND variation (e.g. standard deviation) or associated estimates of uncertainty (e.g. confidence intervals)
☐	☒	For null hypothesis testing, the test statistic (e.g. *F*, *t*, *r*) with confidence intervals, effect sizes, degrees of freedom and *P* value noted<br>*Give P values as exact values whenever suitable.*
☒	☐	For Bayesian analysis, information on the choice of priors and Markov chain Monte Carlo settings
☒	☐	For hierarchical and complex designs, identification of the appropriate level for tests and full reporting of outcomes
☒	☐	Estimates of effect sizes (e.g. Cohen's *d*, Pearson's *r*), indicating how they were calculated

Our web collection on statistics for biologists contains articles on many of the points above.

Software and code

Policy information about availability of computer code

Data collection	No software was used for data collection.
Data analysis	All details of data analysis are described in the Methods. Briefly, open source MAGeCK (version 0.5.6) was used for sequencing analysis of the CRISPR screens, with additional data analyses performed using Python v3.12, with the following packages: pandas (v2.2.2); numpy (v1.26.4); matplotlib (v3.8.4); scipy (v1.12.0). Additional data analysis and visualization was performed using GraphPad Prism (version 10.4.2)Microscopy images were initially captured and processed using ZEN and Zen lite software (Zeiss) prior to final processing with Adobe Photoshop (version 26.7). Figures were created using either Adobe Illustrator (version 27.8.1) or Inkscape (version 1.0.2-2) and were supplemented using images developed with BioRender (BioRender 2024).

For manuscripts utilizing custom algorithms or software that are central to the research but not yet described in published literature, software must be made available to editors and reviewers. We strongly encourage code deposition in a community repository (e.g. GitHub). See the Nature Portfolio guidelines for submitting code & software for further information.

Data

Policy information about availability of data

All manuscripts must include a data availability statement. This statement should provide the following information, where applicable:

- Accession codes, unique identifiers, or web links for publicly available datasets
- A description of any restrictions on data availability
- For clinical datasets or third party data, please ensure that the statement adheres to our policy

All data supporting the findings of this study are available within the paper, its Supplementary Data and Information files, and provided source data. Additional links are provided for raw sequence data associated with library validation and screening outputs are deposited in the Sequence Read Archive (SRA) database under Accession PRJNA1335561 [http://www.ncbi.nlm.nih.gov/bioproject/1335561]. Additional links to plasmid and plasmid libraries associated with the genome-wide screens are deposited at Addgene (#176668 and #234477).

Research involving human participants, their data, or biological material

Policy information about studies with human participants or human data. See also policy information about sex, gender (identity/presentation), and sexual orientation and race, ethnicity and racism.

Reporting on sex and gender	N/A
Reporting on race, ethnicity, or other socially relevant groupings	N/A
Population characteristics	N/A
Recruitment	N/A
Ethics oversight	N/A

Note that full information on the approval of the study protocol must also be provided in the manuscript.

Field-specific reporting

Please select the one below that is the best fit for your research. If you are not sure, read the appropriate sections before making your selection.

☒ Life sciences ☐ Behavioural & social sciences ☐ Ecological, evolutionary & environmental sciences

For a reference copy of the document with all sections, see nature.com/documents/nr-reporting-summary-flat.pdf

Life sciences study design

All studies must disclose on these points even when the disclosure is negative.

Sample size	For CRISPR screening experiments, >1000 cells per sgRNA were represented at all times, a standard number of cell/sgRNA according to previous studies.<br>For mosquito experiments, sample sizes (defined as the number of individual mosquitoes examined) are depicted for each experiment and were determined based on previously published studies in which similar methods were employed..
Data exclusions	No data were excluded from our analysis.
Replication	For CRISPR screening experiments, two replicate screens were performed for both the fitness and clodronate liposome screens with all data reported in the paper. All attempts of replication were successful.<br>All in vivo mosquito experiments were performed with 2 or more independent biological replicates. All attempts of replication were successful. Data were pooled between replicates for analysis.
Randomization	This is not relevant for CRISPR screens were biological experiments were initiated on the same day.<br>Mosquitoes derived from the same batch/cohort were pooled and then used for experiments/treatments for each biologically independent sample. For each experiment, treatments (liposomes, RNAi, etc.) on adults mosquitoes were performed in bulk with individual mosquitoes chosen at random for downstream analysis.
Blinding	CRISPR screening experiments do not include subjective measurements and do not require blinding. For in vivo mosquito experiments, blinding was not possible due to limitations in the number of qualified personnel able to perform experiments.

Reporting for specific materials, systems and methods

We require information from authors about some types of materials, experimental systems and methods used in many studies. Here, indicate whether each material, system or method listed is relevant to your study. If you are not sure if a list item applies to your research, read the appropriate section before selecting a response.

Materials & experimental systems

n/a	Involved in the study
☒	☐ Antibodies
☐	☒ Eukaryotic cell lines
☒	☐ Palaeontology and archaeology
☐	☒ Animals and other organisms
☒	☐ Clinical data
☒	☐ Dual use research of concern
☒	☐ Plants

Methods

n/a	Involved in the study
☒	☐ ChIP-seq
☒	☐ Flow cytometry
☒	☐ MRI-based neuroimaging

Eukaryotic cell lines

Policy information about cell lines and Sex and Gender in Research

Cell line source(s)	Sua-5B (Anopheles coluzzi). A "screen-ready" (attP+ Cas9+) cell line Sua-5B-IE8-Act::Cas9-2A-Neo1 (CVCL_B3N3) was previously published by Viswinatha et al (iPMID: 34819517) and is available from the Drosophila Genomics Resource Center (stock # 334). A library of sgRNAs "GWCRISPKO_AGAM" used for the CRISPR screens is deposited at Addgene (#234477)
Authentication	Details of authentication are described in detail in the Methods section. The sgRNA library was confirmed by sequencing.
Mycoplasma contamination	Cells were negative for Mycoplasma as confirmed by established diagnostic PCR protocols.
Commonly misidentified lines (See ICLAC register)	No commonly misidentified cell lines were used in this study.

Animals and other research organisms

Policy information about studies involving animals; ARRIVE guidelines recommended for reporting animal research, and Sex and Gender in Research

Laboratory animals	Experimental details are described in detail in the Methods section of our manuscript. Briefly, all in vivo mosquito experiments were performed using adult female Anopheles gambiae (Keele strain) mosquitoes. While 21-24g female Swiss Webster mice (Charles River) were used for mosquito infection experiments using P. berghei.
Wild animals	N/A
Reporting on sex	N/A
Field-collected samples	N/A
Ethics oversight	All mouse and in vivo mosquito experiments were respectively approved by the Institutional Animal Care and Use Committee (IACUC) and Institutional Biosafety Committee (IBC) at Iowa State University.

Note that full information on the approval of the study protocol must also be provided in the manuscript.

Plants

Seed stocks	N/A
Novel plant genotypes	N/A
Authentication	N/A
