# Supplementary material for: A genome-wide CRISPR screen in Anopheles mosquito cells identifies fitness and immune cell function-related genes
Source: Nat Commun. 2025 Nov 24;16:10323. doi: 10.1038/s41467-025-65304-y (PMC12644648; doi:10.1038/s41467-025-65304-y)
Supplement: Supplementary file 4 — Description of Additional Supplementary Files [file 41467_2025_65304_MOESM4_ESM.pdf]

Description of Additional Supplementary Files

File name: **Supplementary Data 1**

Description: Summary statistics of the genes targeted by the CRISPR library in each screen and the annotated list of all genes targeted in the library.

File name: **Supplementary Data 2**

Description: sgRNA sequences, read counts, and associated analysis of the fitness screen data.

File name: **Supplementary Data 3**

Description: Summary and comparative analysis of *Anopheles* fitness genes.

File name: **Supplementary Data 4**

Description: *Anopheles* to *Drosophila* gene orthology (GO) mapping and annotations.

File name: **Supplementary Data 5**

Description: Summary and gene list comparisons of Sua-5B expression to previous hemocyte bulk RNA-seq or single-cell RNA-seq studies.

File name: **Supplementary Data 6**

Description: sgRNA sequences, read counts, and associated analysis of the clodronate screen data.
